# Muscle Glycogen in Elite Soccer – A Perspective on the Implication for Performance, Fatigue, and Recovery

**DOI:** 10.3389/fspor.2022.876534

**Published:** 2022-04-25

**Authors:** Magni Mohr, Jeppe F. Vigh-Larsen, Peter Krustrup

**Affiliations:** ^1^Department of Sports Science and Clinical Biomechanics, SDU Sport and Health Sciences Cluster (SHSC), University of Southern Denmark, Odense, Denmark; ^2^Centre of Health Science, Faculty of Health, University of the Faroe Islands, Tórshavn, Faroe Islands; ^3^Section for Sport Science, Department of Public Health, Aarhus University, Aarhus, Denmark; ^4^Sport and Health Sciences, University of Exeter, Exeter, United Kingdom; ^5^Danish Institute for Advanced Study (DIAS), University of Southern Denmark, Odense, Denmark

**Keywords:** football, nutrition, training, carbohydrates, resynthesis

## Abstract

Based on extrapolation of current trends in modern soccer, physiological loading has increased markedly, and the game will continue to become even more demanding in the future, which will exacerbate fatigue at the end of a game and between games. Soccer is a glycogen consuming activity due to its high-intensity intermittent nature, and muscle glycogen is a key factor associated with fatigue late in a game, as well as in determining recovery after a game or an intense training session. Low glycogen in individual muscle fibers and subcellular compartments in the muscle cell is likely to negatively affect several essential steps in the excitation-contraction coupling such as action potential propagation, calcium handling and cross-bridge cycling through reductions in muscle ATP which are suggested sites of muscle function impairment inducing muscle fatigue. Recovery of physical performance and muscle glycogen after a soccer game is a slow process, which challenges the reality in modern elite soccer with increased game and training frequency and physiological loading. We suggest a markedly higher prioritization of fitness training modalities, nutritional approaches and general recovery strategies that optimizes muscle glycogen storage prior to games and training sessions. Also, the soccer community including the governing bodies of the sport must acknowledge and plan according to the high and increasing demands of the modern game, as well as the consequences this has on fatigue and recovery. These aspects are paramount to consider in the planning of training and games, as well as in the process of structuring soccer tournaments and developing competitive regulations in the future to optimize performance and player health.

## Introduction

Soccer is the most popular sport on the global scene with huge participation as well as spectator and commercial interests. The sport has evolved at an accelerating rate over the years and in concert with increased technical and tactical demands it is getting continuously more physically demanding. For example, the number of annual games for a top-class player has increased from 50 to 60 from the 2008–2009 season to the 2018–2019 season, and this number is expected to rise to 70 in 2030 (Nassis et al., 2020). Moreover, the amount of high intensity running and sprinting in games, as well as total weekly training volume has risen immensely over the last decades (see Nassis et al., 2020). These upregulated physiological demands in modern soccer are likely to exacerbate the degree of fatigue, although this could in part be countered by increased fitness preparation and recovery strategies that may itself have driven the intensity upwards. Accordingly, optimization of performance strategies, training modalities, game preparation and recovery regimens before, during and after soccer games are paramount. Although muscle fatigue is considered a multifactorial process with contribution from different fatiguing mechanisms (e.g. metabolic, mental, morphological, biochemical etc.) muscle glycogen depletion has been highlighted as a key factor for end-game exercise tolerance (Mohr et al., 2005). The aim of the present perspective is to present our viewpoint on the importance of skeletal muscle glycogen depletion and resynthesis during and after soccer games and intense training for in-game fatigue development and between-game recovery processes with potential increased impact during periods of multiple games and short recovery. These aspects are of high importance at the present time, but are likely to become an even greater challenge in the future to come.

## Discussion

### Physiological Loading, End-Game Fatigue and Glycogen Depletion in Soccer

Soccer is an intense multicomponent intermittent sport challenging the entire performance spectrum with high concomitant cardiovascular, metabolic, and musculoskeletal fitness demands (Krustrup et al., 2018). This is well-documented by tracking data demonstrating that the top-class players cover 10–13 km in a game of which 2.5 km is classified as high intensity running with the addition of numerous intensive movements such as sprints, accelerations, decelerations, changes of directions, tackles, jumps and shots (Mohr et al., 2005). It should be noted that large inter- and intra-individual variability exists in work rate and physiological loading during games which implies that not all individual players are exposed to the equally high workloads during each game (Fransson et al., 2018). The high game demands in modern soccer is supported by measurements of high peak and average heart rates, elevated blood and muscle lactate levels, increases in plasma free fatty acids, as well as acutely lowered muscle phosphocreatine concentrations after intensive periods, demonstrating a high aerobic and anaerobic energy turnover (Krustrup et al., 2006). This activity pattern and physiological loading provoke fatigue during games, but especially at the final stages, which is evident by a decrement in high intensity running toward the end of a game in concert with deteriorated sprint ability and muscle strength (Mohr et al., 2005). End-game fatigue development has for nearly 50 years been linked with depleted muscle glycogen stores based on early observations of associations between initial muscle glycogen content and physical game performance (Saltin, 1973). More recent studies both in men's (Krustrup et al., 2006) and women's soccer (Krustrup et al., 2021) have demonstrated a substantial game-induced muscle glycogen depletion, especially at the single-fiber level, with two-thirds to three-fourths of individual type 1 and 2 fibers being low on glycogen. Moreover, statistical associations between the degree of muscle glycogen degradation and the decrease in post-game exercise tolerance have accompanied these findings. In support, numerous studies applying dietary carbohydrate manipulations prior to games or game simulations, have demonstrated an indirect relation between muscle glycogen and performance (de Sousa et al., 2021). Finally, strong correlations have been observed between muscle beta oxidative capacity and the ability to maintain performance toward the end of a game (Mohr et al., 2016a), indicative of a performance-enhancing effect of increased fat oxidation, likely due to glycogen sparing.

### Muscle Glycogen and Potential Fatiguing Mechanisms

Despite strong indications of an important role of muscle glycogen in the fatiguing process during high intensity intermittent exercise, such as a soccer game, the exact mechanisms by which reduced muscle glycogen impairs muscle function have not yet been convincingly elucidated. A key aspect may be the heterogeneity in muscle glycogen storage and utilization during high-intensity intermittent exercise with fiber type differences as well differences between single fibers of the same typology (Essen, 1977). Thus, in accordance with the results obtained by Krustrup et al. (2006) and Krustrup et al. (2021), this heterogeneity may result in a high proportion of individual fibers reaching critically low glycogen levels despite the fact that whole-muscle glycogen storage size is not fully exhausted during a 90-min soccer game. In line with this conception, a critical muscle glycogen threshold of ~250 mmol·kg^-1^ d.w. has been proposed, with depletion below this level resulting in impaired exercise tolerance, likely owing to near-depleted single-fiber levels (see Vigh-Larsen et al., 2021). Providing another interesting perspective, glycogen particles are localized in three different cell compartments in proximity to the main energy-consuming steps of the excitation-contraction coupling such as the myosin-ATPase, SR Ca^2+^ ATPase and the Na^+^-K^+^ ATPase (Vigh-Larsen et al., 2021). These distinct subcellular glycogen fractions are similarly utilized in a fiber type and compartment-specific pattern during high-intensity exercise and glycogen degradation in all three subcellular domains have been demonstrated (Nielsen et al., 2012). This may result in perturbations in excitation-contraction coupling function and provide a direct link between reduced muscle glycogen and down-regulation of contractility. For example, intra glycogen (stored within the myofibrils, close to the contractile filaments) has been implicated in reduced SR Ca^2+^ release, whereas IMF glycogen (stored between the myofibrils) has been linked with Ca^2+^ uptake, which have been shown to be negatively affected by a soccer game (Krustrup et al., 2011). Future studies should investigate the effect of low muscle glycogen on other myocellular ion transporters such as the Na^+^-K^+^ ATPase. In addition to the proposed direct associations between muscle function and glycogen depletion, low glycogen also alters muscle metabolism and the systemic homeostasis, which may mediate central fatigue, which is a relatively unexplored but interesting perspective (Vigh-Larsen et al., 2021). Furthermore, exercise-induced muscle damage (EIMD) during eccentric exercise scenarios such as a soccer game may be exaggerated by low muscle glycogen. For example, during a mountain marathon, which has a marked eccentric component, a high intake of carbohydrates (120 g/h) during the race has been shown to attenuate the degree of muscle damage (Viribay et al., 2020). Finally, EIMD and the accompanied elevated inflammatory response, may also attenuate both skeletal muscle GLUT-4 concentration and insulin mediated glucose transport (Asp et al., 1995), which may be a factor affecting recovery of performance and muscle glycogen after a soccer game.

### Implications for Between-Game Recovery

Contrasting a relatively rapid recovery following continuous submaximal exercise, the recovery of muscle glycogen content and performance after a soccer game is a slow-paced process. Thus, ~72 h is required to fully replenish the muscle glycogen stores (Mohr et al., 2005) especially in type 2 fibers (Gunnarsson et al., 2011), which as stated above may relate to microinjuries in the muscle cell, upregulated inflammatory responses and oxidative stress, which are greatly elevated on recovery days in repeated game scenarios (Mohr et al., 2016b). In addition, recovery of performance, muscle soreness and range of motion seem to follow a similar pattern as these parameters (Mohr et al., 2016b). The elite soccer player is, therefore, in a constant race against time to recover from games and high intensity training sessions where efficient muscle glycogen resynthesis appears to be a major recovery component, which may require the combination of several recovery strategies, such as nutritional strategies, cold/hot water immersion, massage and active recovery approaches. Also, an optimal training load between consecutive games is essential. In line with the previously stated increase in game demands, these challenges will only be exacerbated in the future of elite soccer as game and training loads are increasing. This is already evident with the expansion of club and national team tournaments. For example, in the Nations League 2022, European national teams will play four games in a row, with each game only separated by three recovery days. Moreover, the abolishment of the away goal advantage increases the likelihood of more 120-min games, which is expected to aggravate the fatigue responses compared to a regular time game (Mohr et al., unpublished results). As an example of the extreme conditions that individual players can experience, the Liverpool top-star, Mohammed Salah, played four 120-min games over 11 days during the African Nations Cup 2022, when representing his country Egypt. Thus, one of the best players in the world took part in the tournament final, which also went to overtime, following only 3 days of recovery from a game schedule of three consecutive overtime games, providing a very notable recent worst-case scenario example from the world of elite soccer.

### Points to Consider in Future Soccer

Based on the evolution of modern soccer and the discussion above, we have listed 12 points of importance for muscle glycogen, performance, fatigue and recovery in modern elite soccer for men and women.

- The physical demands in elite soccer are evolving rapidly, which is likely to increase the degree of fatigue at the end of and between games.
- Muscle glycogen depletion appears to be involved in the fatiguing process and attaining optimal pre-game concentrations should therefore be a main priority in the planning of training and dietary strategies between games.
- Specific training categories, such as speed endurance production training, has been shown to be effective in increasing the pre-match glycogen stores for type 1 as well as type 2 fibers.
- The training load leading up to games should be controlled and progressively lowered during the last 48 h before a game to allow for muscle glycogen super-compensation.
- Nutritional strategies to ensure an optimal muscle glycogen resynthesis leading up to game, after intense training session and during recovery from games should be prioritized.
- Fitness training should aim also to train muscle beta oxidative capacity, and high intensity interval training has been shown to be an efficient training modality targeting mitochondrial function and beta oxidation.
- The use of all five substitutions that has been allowed in international soccer should be utilized more systematically especially during the last 30 min of a game.
- Rotation of players with extraordinary high game demands should be applied in situations with repeated match-play exposure.
- Tactical adjustments should be considered to reduce the game intensity in scenarios with repeated match-play exposure.
- Supply of carbohydrates should be considered after the warm-up, at half-time and potentially in breaks during the game – these aspects are paramount in 120-min matches and repeated game situations.
- Strength/power training with an included eccentric component should be performed on a regular basis to decrease the degree of muscle damage during games and intense training sessions.
- Finally, the soccer governing bodies should consider the increased demands and challenging competitive conditions that currently are present in elite soccer when planning international and domestic tournaments. Factors such as squad sizes and rules for number of substitutions, as well as duration between games are important aspects of these considerations.

## Data Availability Statement

The original contributions presented in the study are included in the article/supplementary material, further inquiries can be directed to the corresponding author.

## Author Contributions

MM, JFV-L, and PK contributed substantially to the conception and design of the article. All authors drafted the manuscript and revising the different versions critically for important intellectual content. All authors approved the manuscript for publication and are accountable for all aspects of the work in ensuring accuracy or integrity of any part of the work.

## Funding

The work done was supported by Novo Nordisk 570 Foundation grant to Team Denmark (PRoKIT research network), as well as the Faroese Research Council.

## Conflict of Interest

The authors declare that the research was conducted in the absence of any commercial or financial relationships that could be construed as a potential conflict of interest.

## Publisher's Note

All claims expressed in this article are solely those of the authors and do not necessarily represent those of their affiliated organizations, or those of the publisher, the editors and the reviewers. Any product that may be evaluated in this article, or claim that may be made by its manufacturer, is not guaranteed or endorsed by the publisher.
